# Features of Oxidative and Nitrosative Metabolism in Lung Diseases

**DOI:** 10.1155/2019/1689861

**Published:** 2019-05-22

**Authors:** Svetlana Soodaeva, Nailya Kubysheva, Igor Klimanov, Lidiya Nikitina, Ildar Batyrshin

**Affiliations:** ^1^Pulmonology Scientific Research Institute under FMBA of Russia, Orekhovyy Bul'var 28, Moscow 115682, Russia; ^2^Kazan Federal University, Kremlyovskaya St., 18, Kazan 420000, Russia; ^3^Khanty-Mansiysk-Yugrа State Medical Academy, Mira St., 40, KMAD-Yugry, Khanty-Mansiysk 628007, Russia; ^4^Centro de Investigación en Computación, Instituto Politécnico Nacional (CIC-IPN), Av. Juan de Dios Bátiz, Esq. Miguel Othón de Mendizábal S/N, Gustavo A. Madero, 07738 Mexico City, Mexico

## Abstract

Respiratory diseases are accompanied by intensification of free radical processes at different levels of the biological body organization. Simultaneous stress and suppression of various parts of antioxidant protection lead to the development of oxidative stress (OS) and nitrosative stress (NS). The basic mechanisms of initiation and development of the OS and NS in pulmonary pathology are considered. The antioxidant defense system of the respiratory tract is characterized. The results of the NS and OS marker study in various respiratory diseases are presented. It is shown that NS and OS are multilevel complex-regulated processes, existing and developing in inseparable connection with a number of physiological and pathophysiological processes. The study of NS and OS mechanisms contributes to the improvement of the quality of diagnosis and the development of therapeutic agents that act on different pathogenetic stages of the disease.

## 1. Introduction

Most diseases of the respiratory tract (RT) are accompanied by the intensification of free radical processes at different levels of the biological organization of the body with simultaneous stress and subsequent oppression of various links of antioxidant protection, which leads to the development of oxidative stress (OS)—imbalance in the reactive oxygen species (ROS) and antioxidant protection of the body [1–3].

Over the past decade, much attention has been paid to studying the molecular mechanisms of the development of both oxidative stress and nitrosative stress (NS) in lung diseases, as well as the identification of prognostic and diagnostic markers in various biological media and the elucidation of the possibilities of therapeutic influence on OS and NS. These processes are inherently associated with the development and course of inflammatory and other physiological and pathophysiological mechanisms that are pathogenetic links in the development of the disease. The initiation of OS and NS can occur by an exogenous and/or endogenous pathway [2, 4–7].

## 2. Activation of Oxidative and Nitrosative Stress in the Respiratory Tract

For the respiratory tract, the exogenous pathway of the OS and NS initiation is the most relevant. So, about 8000 liters of air containing various gases (oxygen and volatile oxides), infectious agents (bacteria, viruses, and fungi), pollutants, and allergens, which have prooxidant effects, passes through the lungs every day. The main air pollutants of the urban atmosphere are particulate matters (PM), which are a variable composition of organic and inorganic compounds with a carbon core. OS-induced air pollutants and damage to the respiratory tract occur with the participation of metals of variable valency, trace amounts of which are part of PM. In addition to the initiation of the OS and NS by prooxidants, free radicals can be also in significant amounts in the inhaled air. The gas phase of tobacco smoke contains about 1015 free radicals in one puff, including superoxide anion and hydroxyl radicals. Among the exogenous factors of the OS initiation, short-wave electromagnetic radiation (UV, X-rays, etc.) should also be considered [8, 9].

The endogenous pathway of the OS and NS initiation is represented by a wide variety of mechanisms. The redox reactions accompany a huge number of biochemical processes in vivo. One of the main intracellular sources of free radicals is mitochondrial respiration: 1-2% of electrons can “leak” from the respiratory chain [9]. Radicals and other highly active oxidants are formed in various ways. There are so-called primary radicals which are formed by an enzymatic way: superoxide anion radical and nitric oxide. These radicals give rise to such two pools of highly active groups of molecules as reactive oxygen species (ROS) and reactive nitrogen species (RNS).

**Figure 1:**
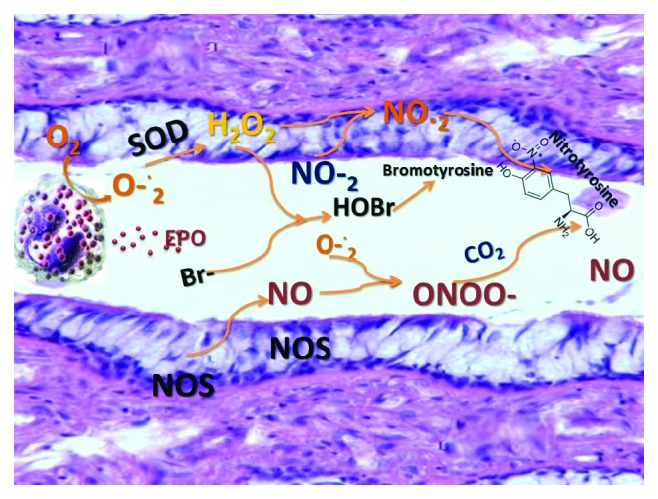
ROS and RNS formation in the respiratory tract.

The division into ROS and RNS is rather conditional since, in biochemical processes, the radical and nonradical forms of these compounds react with each other. Primary radicals, interacting with various compounds from their microenvironment, form secondary radical, tertiary radical, and so on; highly active nonradical forms; and stable products (Figure 1). ROS includes superoxide anion radical (O_2_^·−^), hydroxyl radical (ОН^·^), peroxyl radical (НО_2_^·^), and alkoxy radical (RO). During the reaction, ROS derivatives are formed, such as hydrogen peroxide (H_2_O_2_) and lipoperoxides (ROOH). RNSs include nitric oxide (NO), other higher nitrogen oxides, nitrites, and peroxynitrite (ONOO^−^). Oxidases are involved in the generation of superoxide anion radical: NADPH oxidase, xanthine oxidase, cytochrome P-450 oxidase, etc. [2, 10]. The formation of NO occurs with the help of NO synthase enzymes (NOS) in the NO cycle and with the participation of nitrite/nitrate reductase systems [11].

**Figure 2:**
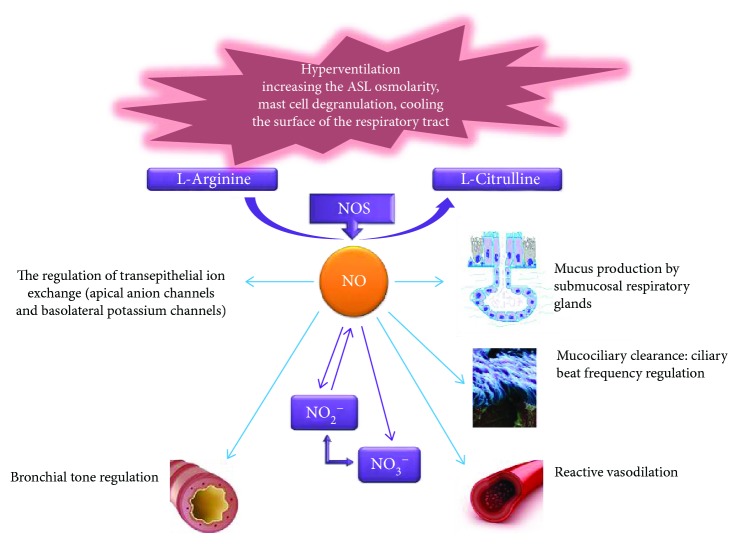
Bronchoprotective NO properties. ASL: airway surface liquid.

The physiological role of NO in the respiratory tract (Figure 2) is also widely known. It includes regulation of the basal tone and vascular permeability, modulation of bronchial reactivity, and antimicrobial protection. NO is able to regulate the secretion of bronchial mucus produced by glands located in the submucosal layer of the bronchi. Nagaki et al. studied the effect of inhibitors of NO-synthase L-NAME and L-NMMA on the secretion of mucin glycoproteins by determining glycoconjugates precipitated with trichloroacetic acid in the explants and isolated human submucosal glands [12]. NO synthase inhibitors have been shown to have no direct effect on the secretion of glycoproteins, suppressing the secretion of methacholine and bradykinin in isolated glands. In addition, isosorbide dinitrate, as a source of NO, contributed to a significant increase in mucin secretion. The results of this study suggest a stimulating effect of endogenous NO on the mucin production by the submucosal glands of the respiratory tract.

NO synthase inhibitors slow down the frequency of ciliary beats of respiratory epithelial cells of cows stimulated with isoproterenol, bradykinin, and substance P. This effect is completely reversible when adding the precursor of NO L-arginine, which indicates the NO-dependent mechanism of ciliary motor stimulation by the above-named compounds. Ciliary motility is also activated by TNF*α* and IL-1*β*, produced by alveolar macrophages (inducible NO synthase pathway). This stimulating effect is blocked by L-NMMA and restored with the addition of L-arginine, confirming the regulatory role of inducible NO-synthase in its implementation [12].

In addition to the activity of ciliary motility, the effectiveness of mucociliary clearance is determined by the properties of the airway surface liquid (ASL), the composition and volume of which, in turn, depends on the transport of electrolytes. The functional activity of ion channels is also highly susceptible to the modulating effect of NO. The NO molecule activates both the apical anion channels and the basolateral potassium channels along the cGMP-dependent pathway, acting as a physiological regulator of transepithelial ion exchange [12, 13].

The ability of endogenous NO to modulate bronchial hyperreactivity (BHR) induced by various mediators has been experimentally confirmed. Nijkamp et al. revealed histamine-induced bronchoconstriction in guinea pigs by the inhibition of NO synthase in vivo, as well as a dose-dependent reduction under the action of histamine of the smooth muscles of the tracheal tube of the guinea pig in vitro [13]. Ricciardolo et al. showed NO-dependent regulation of bronchoconstriction induced by bradykinin, citric acid, selective NK1 tachykinin agonist, and protease-activated receptor 2 in guinea pigs [14, 15].

Intraluminal perfusion of the intact tracheal tube of guinea pigs with bradykinin, endothelin-1, substance P, adenosine, and calcitonin with a gene-bound protein led to dose-dependent relaxation [14, 15]. At the same time, the addition of the NO synthase inhibitor was accompanied by a tracheal tube contraction, which confirms the NO-dependent mechanism of respiratory tract relaxation. The same effect was reproduced by removing the respiratory epithelium. Consequently, the respiratory epithelium is the main source of endogenous NO, which prevents bronchoconstriction under the influence of various triggers. The results of the study emphasize the significant role of the respiratory epithelium in the BHR regulation. It is not just a physiological barrier between bronchoconstrictive stimuli and smooth myocytes but also a modulator of the bronchial tone through the release of epithelial relaxation factors.

Further research demonstrated a rapid (within 2 seconds) release of NO in the respiratory epithelium of guinea pigs induced by bradykinin. This phenomenon was absent in the subepithelial layer, free from calcium ions [14, 15]. Therefore, the endogenous bronchoprotective NO release occurs with the participation of calcium-dependent constitutive NO synthase.

An additional mechanism NO bronchoprotective effect in the airways is the cGMP-dependent property of smooth bronchial myocytes. Thus, bradykinin-induced increase in cGMP production in the guinea pig airways was demonstrated. This effect was blocked by the addition of NO synthase inhibitors, which indicates the role of cGMP as a final mediator of NO-dependent epithelial bronchoprotection [14, 15].

In vitro and in vivo studies have shown that BHR, caused by allergen exposure, does not increase with the preliminary addition of NO synthase inhibitors. The BHR-induced virus is completely blocked during exposure to L-arginine, which demonstrates the interrelation of this syndrome with the lack of endogenous NO. It has also been established that a deficiency in constitutive NO synthase production in guinea pigs leads to the BHR progression as part of an early allergic reaction (4-6 hours after allergen exposure), and the restoration of the NO level with inducible NO synthase contributes to the reverse BHR development during later timing (24-48 hours). These conclusions were made based on ineffective inhalation of a specific inhibitor of inducible NO synthase aminoguanidine on histamine-induced BHR after an early allergic reaction and significant BHR decrease after inhalation during the late allergic response [14, 15].

Moreover, Toward and Broadley found that lipopolysaccharide inhalation by guinea pigs suppressed respiratory NO production, which followed by an increase in histamine-induced hyperreactivity (one hour after exposure). 48 hours after inhalation, BHR to histamine decreased simultaneously with an increase of NO metabolites in bronchoalveolar lavage, suggesting a resumption of NO synthesis when activating the NF-*κ*B-dependent expression of the inducible NO synthase [16].

The above NO-dependent mechanisms of bronchodilation, activation of mucociliary clearance, and bronchoprotective properties of NO become crucial during exercise with increase minute ventilation.

In addition to direct registration in exhaled air, the production of NO in RT can be determined from the concentration of its more stable metabolites, such as nitrate and nitrite anions, 3-nitrotyrosine, and nitrosothiols in the exhaled breath condensate (EBC). Nitrate and nitrite anions are the most stable of these metabolites. The metabolism of nitric oxide and oxygen radicals has common points of contact; therefore, it is necessary to emphasize the importance of simultaneously evaluating several indicators of molecular metabolism in EBC for a more objective interpretation and interpretation of results.

In recent decades, considerable evidence about the contribution of NO and its metabolites to the pathogenesis of many diseases of the respiratory tract has been accumulated. So, the total concentration of nitrates and nitrites (TNN) in EBC is significantly increased in patients with uncontrolled and controlled bronchial asthma compared with healthy children [17, 18]. In addition, in patients with absolute and complete control of bronchial asthma who had not received therapy by glucocorticosteroids, the total content of nitric oxide metabolites in EBC was significantly higher than in patients receiving this therapy. There was established a significant relationship between the total content of nitric oxide metabolites in EBC and the level of control of bronchial asthma in patients treated with the same type of therapy (including glucocorticosteroids).

The EBC concentration of 3-nitrotyrosine (3-NT) is higher in patients with controlled mild asthma not receiving corticosteroids, but it is lower compared with the moderate and severe asthmatics, using inhaled corticosteroids. The level of 3-NT correlates with the level of fractional exhaled NO (FeNO) only in controlled mild asthma [17, 19]. The level of nitrosothiols was studied in patients with mild and moderate asthma. An increase in this parameter was noted in patients with moderate asthma compared with controls and patients with mild asthma [17, 20].

The results of the studies of the exhaled NO level in chronic obstructive pulmonary disease (COPD) are contradictory. Kubysheva et al. demonstrated that the severity of the progression of COPD was linked with an increase in the concentrations of metabolites of nitric oxide in blood and in exhaled breath condensate [21, 22]. For the patients with COPD, the associations between the lung function parameters and the levels of ΣNO_2_^−^/NO_3_^−^ were determined.

However, it was found that smoking and disease severity are the most important factors affecting NO production [23]. Active smokers with severe COPD (especially in combination with a pulmonary heart) show lower levels of exhaled NO compared with former smokers with a nonsevere COPD. An increase in FeNO has been reported in patients hospitalized with exacerbation of COPD. FeNO of patients receiving systemic corticosteroids returned to control values only months later after discharge from hospitals. This result confirms various mechanisms of inflammation in COPD and steroid-sensitive asthma. Acidosis, which often accompanies acute ventilatory respiratory failure associated with an exacerbation of COPD, may also contribute to an increase in exhaled NO [24].

Other disorders associated with the activation of NS include bronchiectasis, active pulmonary sarcoidosis, active pulmonary fibrosis, and the transplant rejection reaction of the lungs [24].

In cystic fibrosis (CF) remission, there is an increase in EBC nitrite anion [25], in contrast to the FeNO level [26]. Formanek et al. [25] showed an increase in nitrate anion and 3-NT with normal FeNO levels in sputum of patients with CF. Increased production of NO and superoxide anion radical may not be accompanied with increase NO in EBC since the constant for the reaction of superoxide with NO is higher than the constant for its reaction with SOD [27, 28].

The role of human microbiota in the nitric oxide cycle, the role of significant components of nitrite and nitrate-reductase systems in the nitric oxide cycle, and the mechanisms of their activation and deactivation (participation of enzymes, cofactors, homeostatic indicators, etc.) are determined under various conditions, which allows detailed control mechanisms of the NO cycle for targeted exposure to therapeutic agents [11].

Recently published data demonstrate the potentially positive role of NOS inhibitors in the treatment of asthma and COPD [29–32]. Inflammation and oxidative stress contribute to the reaction of superoxide anion with available NO, rather than its endogenous neutralizer superoxide dismutase, thus increasing the formation of peroxynitrite in tissues [29, 33]. Consequently, the use of iNOS inhibitors may restore the preferred pathway of superoxide radical detoxification, via superoxide dismutase [34]. Moreover, oxidative and nitrosative stress contribute to arterial stiffness pathogenesis due to oxidative damage to lipids, proteins, and DNA in endothelial cells and uncoupling of NO synthase, leading to endothelial dysfunction. Thus, another target for NOS inhibitors in COPD is arterial stiffness elevation by nitrosative stress, which accompanies structural local and systemic changes in those patients [30]. During rhinovirus-induced COPD exacerbation, high levels of reactive nitrogen species induce nitrosylation of histone deacetylases-2 (HDAC2) and reduced HDAC2 activity in macrophages. This is believed to be a key mechanism of corticosteroid resistance in COPD and can be modified by NOS inhibitors as well [31].

There is evidence of the potential role of the NO donor in the treatment or modulation of asthma and COPD in viral exacerbations [35–38]. Rhinovirus-infected epithelial cells are known to produce chemotactic cytokines, which attract inflammatory cells to airways of patients with asthma and COPD [38]. At the same time, Sanders et al. [5, 39] showed that respiratory epithelial cells also produce nitric oxide (NO), which can play an important role during the antiviral response. NO can inhibit both the replication of the rhinovirus and the rhinovirus-induced production of cytokines in human respiratory epithelial cells [39]. In addition, there is evidence that the addition of chemical NO donors or the induction of NOS causes inhibition of a wide range of viruses, including both DNA and RNA viruses [40]. In vitro studies showed the ability of NO to inhibit human rhinovirus- (HRV-) induced production of IFN-gamma-inducible protein 10 (CXCL10) by inhibiting viral activation of nuclear factor kappa B (NF-kappaB) and of interferon-regulatory factors (IRF), including IRF-1, through a cGMP-independent pathway [41]. Sanders et al. also showed that NO donor 3-(2-hydroxy-2-nitroso-1-propyl-hydrazino)-1-propanamine (NONOate) inhibits the HRV-induced GM-CSF mRNA levels [42]. Thus, NO may play an important antiviral role, and nitric oxide donors may represent a new therapeutic approach for viral exacerbations of asthma and COPD.

## 3. Markers of Oxidative and Nitrosative Stress in Lung Diseases

The study of RNS and ROS concentrations in in vivo and in clinical practice, in particular, is very problematic, due to the specificity of the detected compounds. The lifetime of most ROS and RNS is hundredths of a second or less. Accordingly, a number of requirements of practical properties, characterized by ease of use and reproducibility of the results, are imposed on both the methods and the biological media in which the dynamic concentration of oxidants is monitored. In this regard, noninvasive techniques and biological media have the advantage of studying ROS and RNS in the respiratory tract: exhaled breath condensate (EBC) and exhaled air itself. EBC is a liquid formed as a result of cooling and subsequent condensation of exhaled air; therefore, its composition is determined by the composition of exhaled air. To determine the respiratory, ROS use indicators in the blood, which is, as a rule, a reflection of systemic changes in the redox status in the body. EBC and exhaled air markers help to identify the tension of oxidative and nitrosative status directly in the respiratory tract and the airway surface liquid, which is essentially the first line of lungs and whole-body protection from exogenous oxidants. Noninvasive methods of ROS and RNS studying can optimize diagnosis and treatment, as well as help clarify the molecular mechanisms of the pathogenesis of lung diseases [2, 14, 43].

Among the identified OS markers, conditionally stable RNS and ROS and their metabolism products, other oxidation products of RNS and ROS, and ions of variable valency are the most popular in clinical practice [2, 14, 43].

Despite the difficulties in determining the concentration of certain compounds in the EBC, researchers are now increasingly interested in identifying molecules involved in the reactions of the OS and NS, which are key elements in the development of most pulmonary pathologies, allows assessing noninvasively the state of the respiratory tract. Practically, all these molecules are united by the fact that their concentrations in EBC vary from micromolar to nanomolar, and this, in turn, requires the use of highly sensitive determination methods. Despite the high variability of studied parameters, by increasing the samples, statistically significant differences between groups are achieved in diseases such as asthma, COPD, and CF. Among the most intensively studied molecules, a special place is occupied by the markers of OS and NS: ROS and RNS. However, taking into account the specifics of EBC collection, namely, the duration of the procedure from 10 to 20 minutes and the possible change in the concentration of the molecules being determined during this time, the most promising role is assigned to stable metabolites of oxygen and nitrogen [2, 14, 43].

The most studied form of the ROS is hydrogen peroxide (H_2_O_2_). Hydrogen peroxide in vivo is the product of the superoxide anion radical (О_2_^−^) dismutation. The sources of such radicals are reactions involving xanthine oxidase and mitochondrial and microsomal electron transfer chains. The concentration of H_2_O_2_ in the inflammatory regions is especially high, due to which a change in the H_2_O_2_ level in biological fluids is one of the inflammatory markers [17]. However, the final concentration of H_2_O_2_ in tissue depends on many parameters. Thus, the concentration of any substance in the body at a given time, including hydrogen peroxide, is the sum of the synthesis rate and the rate of decomposition of this compound. When H_2_O_2_ is formed in the dismutation reaction of superoxide with the participation of SOD, the reaction rate constant is lower than during the interaction of superoxide and nitric oxide, which indicates the competition between NO and SOD [44]. Therefore, in the presence of NO, the content of which increases in the focus of inflammation according to many studies; the concentration of hydrogen peroxide in the tissue may decrease. For example, the work of Latzin and Griese [45] revealed a negative significant correlation between the levels of atmospheric NO, and in FeNO, and the content of hydrogen peroxide in EBC in 102 healthy children. The presence in the environment of metal ions of variable valency, such as iron, copper, and manganese, can also reduce the H_2_O_2_ content even at high rates of its formation. This is due to the high rate of decomposition of H_2_O_2_ in the Fenton reaction with the formation of an extremely reactive hydroxyl radical [17].

Changes in the H_2_O_2_ concentration can also be observed when the antioxidant (AO) status changes, namely, medium levels of catalase, peroxidase, peroxiredoxins, and other enzymes that have a high affinity for hydrogen peroxide. Such a high dependence on the concentration of other molecules present in the medium makes it necessary to measure along with hydrogen peroxide a number of parameters: the concentration of metal ions of variable valency (iron, copper, and manganese), the level of nitric oxide (NO), and AO status of the organism [17].

Currently, the dynamics of changes in H_2_O_2_ in EBC has been studied in many pathologies of the respiratory tract. In asthma, an increase in the concentration of H_2_O_2_ in EBC was shown to correlate with an increase in the level of sputum eosinophils and serum content of eosinophilic cationic protein, as well as with a decrease in FEV1 [46, 47]. It is reported that a significant increase in the H_2_O_2_ content in EBC, observed in patients with moderate and severe asthma, can serve as an informative marker of the inflammation severity, unlike the FeNO level, which is highly dependent on the therapy (in particular, corticosteroids) [47]. The possibility of monitoring the asthma therapy efficacy by the estimation of the H_2_O_2_ level in the EBC is shown. In other inflammatory diseases of the respiratory tract, such as COPD [48–50] and bronchiectasis [51], an increase in the H_2_O_2_ level in EBC is also observed. Increased H_2_O_2_ concentrations have also been observed in patients with pneumonia. A positive significant correlation was noted between the level of H_2_O_2_ and the content of thiobarbituric acid (TBA) in EBC on days 1 and 3 of the disease, as well as between the level of H_2_O_2_ in EBC and the concentration of C-reactive protein and the level of blood leukocytes on day 1. During the therapy, a decrease in OS parameters in EBC was revealed [52].

Those H_2_O_2_ level dynamics in EBC with these diseases may indicate an increase in the superoxide generation by cells involved in the inflammation process [53, 54] and/or a decrease in AO activity of cells and tissues with the progression of these pathologies. However, in the group of patients with stable CF, no statistically significant change in this parameter was found in comparison with the healthy control group [55].

F2-isoprostanes (F2-IsoPs) are recognized as reliable markers of oxidative stress in lung diseases [56, 57]. IsoPs have been elevated in plasma, urine, bronchoalveolar lavage in patients with mild and severe asthma [58, 59], interstitial lung diseases [60], cystic fibrosis [61], and chronic obstructive pulmonary disease [62].

Development of sensitive and special radioimmunoassay (RIA) allowed measuring the content of 8-isoprostane, which is an isomer of prostaglandin-F2A, in the exhaled air condensate [63]. An increase IsoP concentrations in EBC was also recorded in children with asthma and persistent allergic rhinitis [64], in smokers with COPD [62], and in healthy volunteers after exposure to ozone [65].

IsoPs are not only markers of oxidative stress but also play an important role in lung pathophysiology. In the respiratory tract, isoprostanes are able to regulate cellular processes that affect the tone of pulmonary vessels and smooth muscles of the airways [66] and to stimulate the adhesion and function of macrophages [67]. Arezzini et al. demonstrated the profibrotic role of F2-IsoPs in the pathogenesis of pulmonary fibrosis. In this study, it was shown that F2-IsoPs can activate fibroblasts in myofibroblasts and participate in the synthesis of collagen [68]. These findings provide evidence of the role of isoprostanes in airway inflammation and, thus, identify a potential therapeutic goal for the treatment of pulmonary diseases.

The main airway biomarkers of the oxidative and nitrosative stress in asthma and COPD are summarized in Table 1.

## 4. Respiratory Antioxidant Protection System

Due to the huge number of reactions occurring in cells and the formation of RNS, ROS, and other highly active compounds, antioxidant (AO) systems exist to regulate the redox balance. The components of these systems are variously distributed both in the cell and at the organ-tissue level. The respiratory tract, which is the first line of body defense against the effects of atmospheric pollutants [2], contains a large number of AO systems. Antioxidant protection of the lungs and airways is carried out by many low molecular weight antioxidants; however, AO enzymes play the main role in protecting the trachea, bronchi, and alveoli epithelium from oxidative damage. The most important AO enzymes include superoxide dismutases (SODs), catalase, glutathione peroxidase (GPx), glutathione S-transferases (GSTs), glutamylcysteine synthases (GCSs), glutaredoxins (Grxs), and thioredexins (Trxs). In humans, all these AO enzymes have been shown to be expressed in the airways [2, 85, 86].

Superoxide dismutases (SODs) are one of the main AO enzymes that are expressed in almost all cells of the human body [87]. The proteins of this family implement the dismutation reaction of O_2_^·−^ to H_2_O_2_. The active center of SOD contains a transition metal ion. For the cytoplasm of eukaryotes, these are copper and zinc ions (Cu/Zn-SOD) [88, 89]. Mitochondria, for example, contain another type of SOD, which includes manganese (MnSOD) in the active center [90]. The rate of enzymatic dismutation of O_2_^·−^ is very high (the rate constant is about 10^9^ M^−1^ s^−1^); it is necessary for the rapid control of the O_2_^·−^ formation. However, the O_2_^·−^ dismutation reaction can proceed more slowly and spontaneously without the participation of the enzyme. Excessive O_2_^·−^, at the same time, can react with a variety of cellular targets and impair their functions. For example, the enzyme of the tricarboxylic acid cycle aconitase is inactivated by O_2_^·−^, which can cause significant changes in cell metabolism [91]. So, generated H_2_O_2_ as a result of the O_2_^·−^ dismutation reaction, like other ROS, can be toxic to cells and its concentration in cells is also controlled by AO systems.

Catalases are heme-containing enzymes that catalyze the decomposition of H_2_O_2_ to H_2_O and molecular oxygen. Glutathione peroxidase (GPx) is another group of proteins involved in the removal of H_2_O_2_. The reduction of H_2_O_2_ to H_2_O, carried out by this group of enzymes, is associated with the oxidation of glutathione. In addition to H_2_O_2_, GPx can interact with other peroxides in cells [92].

The thioredoxin system includes thioredoxin (Trx) itself and thioredoxin reductase (TrxR). Thioredoxins form a family of small-sized proteins with oxidoreductase activity. Trx restores oxidized disulfide peptides under OS. The reduction of oxidized Trx, in turn, is carried out by NADPH-dependent thioredoxin reductases (TrxRs) [93]. In addition to thioredoxin, TrxR can reduce a large number of other compounds. Thus, TrxR1 is involved in the reduction of H_2_O_2_ and lipid hydroperoxides formed in high concentrations at OS. The functions of the thioredoxin system overlap with the glutaredoxin-dependent system, which is also of great importance for AO protection. The component of this system is glutaredoxin (Grx), which is involved in thiol/disulfide exchange reactions. The oxidized Grx is reduced nonenzymatically by the reduced glutathione (GSH) pool. Oxidized glutathione (GSSG) restores a special enzyme glutathione reductase (GR). GR has some similarities with TrxR and is a FAD-containing enzyme that uses reducing equivalents of NADPH to restore GSSG [94].

**Figure 3:**
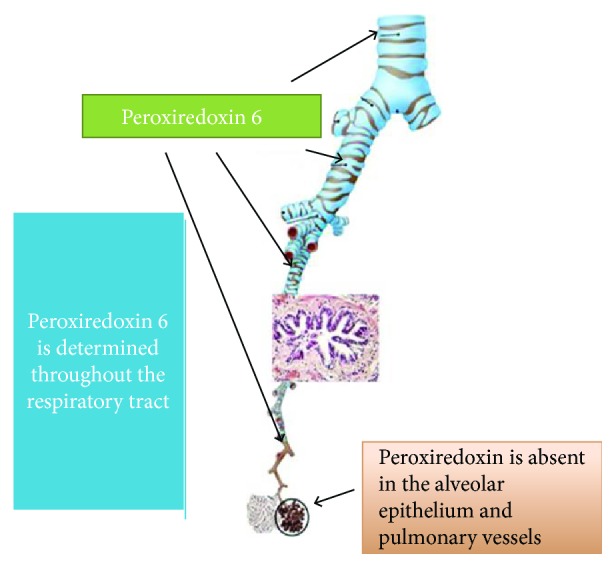
Localization of peroxiredoxin 6 in the lungs.

Peroxiredoxins, a family of AO proteins with peroxidase activity, are also involved in H_2_O_2_ degradation. They were first found in yeast and then in other organisms [85, 86]. In mammals, there are 6 types of peroxiredoxins (Prx1-Prx6), and most of them have been identified in databases recently. In the cell, the peroxiredoxins are found mainly in the cytosol, as well as in the mitochondria, peroxisomes, and chloroplasts. The two isoforms of peroxiredoxins (Prx4 and Prx6) are secretory proteins [85]. The concentration of peroxiredoxins in many cells is unusually high—from 0.1 to 1% of the total water-soluble cellular protein, depending on the type of tissue, making them a redox buffer that controls the level of intracellular peroxides. Peroxiredoxins catalyze the reduction of H_2_O_2_ and organic peroxides to water and alcohol, respectively. In addition, some isoforms of peroxiredoxins can destroy peroxynitrite. Peroxynitrite reductase activity was first detected in bacterial peroxiredoxins and then confirmed for eukaryotic peroxiredoxins [85]. Neutralization of peroxides and peroxynitrite with peroxiredoxins occurs according to the same catalytic mechanism. The expression level of genes of various types of peroxiredoxins is significantly increased in many pathological conditions accompanied by OS. This correlation indicates that cells increase AO protection with peroxiredoxins to neutralize the increased content of ROS. Peroxiredoxin 6 (Prx6) is one of the types of peroxiredoxins that performs a special function in the respiratory tract (Figure 3). The secretory water-soluble Prx6 was first isolated from the rat olfactory epithelium at the Institute of Biophysics of the Cell of the Russian Academy of Sciences [85, 86]. Biochemical studies have shown that Prx6 in the presence of some thiols has the ability to neutralize both organic and inorganic peroxides and its protective activity is determined mainly by peroxidase activity. Immunohistochemical studies conducted at the light and electron microscopic levels, and in situ hybridization experiments have shown that Prx6 is mainly localized in the olfactory epithelium, bronchi, and epidermis. In the trachea and bronchi, it is synthesized by the goblet cells and the Clara cells and secreted into the mucus, where it is major among the water-soluble mucus proteins. Finally, it was shown that the contribution of Prx6 to the neutralization of ROS in the trachea and bronchi reaches 70% [85, 86].

The important role of Prx6 in the protective mechanisms of the bronchial epithelium which primarily include the activation of the Prx6 expression under various pathological conditions led to the assumption that the use of exogenous Prx6 can significantly accelerate the recovery of the affected respiratory epithelium. This hypothesis was tested on models of the acute inflammatory process in the trachea of the rat, caused by bacterial endotoxins, and thermal burns of the upper respiratory tract [85, 86].

The study of AO enzymes in human lung pathology is hampered by a number of objective factors, namely, the inability to assess in vivo protein expression by immunohistochemical methods, the absence of large molecules in noninvasive biological media, the invasiveness of procedures for obtaining biopsy tissue of the lung, the lack of standardization for the evaluation of AO enzymes in the BAL, and induced sputum. The study of the role of enzyme AO systems is currently being evaluated in the blood, in model systems, and in animals. Due to the fact that the work of the AO systems often occurs site-specific (directly at the site of the development of the OS or NS), the evaluation of the AO systems in blood gives conflicting results.

Various low molecular weight substances play the role of antioxidants. These include, in addition to glutathione, ascorbic acid, *α*-tocopherol, and some others [95–98].

The *α*-tocopherol molecule (vitamin E) consists of a benzene core with a hydroxyl group (capable of giving an electron, performing the AO function) and a side wick chain that performs the hydrophobic interaction of the antioxidant with membrane structures. Vitamin E is able to quench ROS, interact with the hydroxyl radical, and restore the lipid radicals of the structure R^·^ and ROO^·^. The most active in the lipid bilayer *α*-tocopherol restores peroxyl radicals. The resulting *α*-tocopherol radical is relatively little active due to the delocalization of the unpaired electron along the aromatic ring. It is believed that in the presence of water-soluble antioxidants, such as the reduced form of ascorbic acid, vitamin E is able to restore its AO potential through direct recycling. Retinol (vitamin A) in combination with *α*-tocopherol is also involved in the protection of biological membranes from damage by their prooxidants [95–98].

Ascorbic acid (AA) is an important representative of water-soluble antioxidants. The presence of two-phenolic groups in the AA molecule structure allows it to participate in redox transformations, acting as a donor and acceptor of electrons and protons. AA has an extremely wide range of AO properties related to hypohalides, О_2_^−·^, НО_2_^·^, RO_2_^·^, 1O_2_, HO^·^, NO^·^, ONOO^−^, and nitrosamines. It also restores the oxidized form of *α*-tocopherol. In the presence of metal cations of variable valency, AA becomes a powerful prooxidant, which is a reliable regulation of the transition metal ion concentration [95–98].

N-Acetylcysteine (NAC), an acetylated amino acid L-cysteine, inactivates free radicals and reactive oxygen species by directly reacting with them (direct antioxidant effect) and also supplying cysteine and promotes the synthesis of glutathione (indirect antioxidant effect). In turn, glutathione is an important component of the detoxification system of xenobiotics, peroxide compounds, and free radicals, which has a protective effect at the cellular level [99, 100].

Of the three amino acids involved in the structure of glutathione (glutamate, glycine, and cysteine), cysteine has the lowest intracellular concentration. At the same time, the main mechanism of glutathione replenishment is de novo synthesis. Consequently, cysteine efficiency may limit the rate of glutathione synthesis under conditions of oxidative stress.

NAC has been used in clinical practice for over 50 years. The positive effect of NAC has been demonstrated on conditions characterized by decreased production of glutathione (GSH) or activation of lipid peroxidation (POL): tobacco smoking, cardiovascular diseases [101], acetaminophen (paracetamol) [102] and heavy metal poisoning, HIV infection, etc. [99, 100]. Preliminary studies demonstrate the effectiveness of NAC as a chemoprophylactic agent in chemotherapy of malignant tumors. The additional roles of the drug are the eradication of Helicobacter pylori and the prevention of gentamicin-induced hearing loss in patients on hemodialysis [103–105].

The prevention of glutathione reserves depletion under the NAC action, and the direct neutralizing effect of the drug in relation to ROS and RNS (OH, H_2_O_2_, ONOO^−^, and O_2_^-·^) helps to reduce the intensity of reactions of OS and NS, which leads to the suppression of inflammation in chronic obstructive pulmonary disease (COPD), influenza, and idiopathic pulmonary fibrosis [100, 101].

COPD is the leading cause of death and morbidity worldwide and is characterized by persistent airflow restriction, hypersecretion and an increase in sputum viscosity, OS, chronic respiratory inflammation, and extrapulmonary manifestations. Currently, a significant evidence base has been collected on the positive impact of NAC on the course of COPD. The antioxidant and anti-inflammatory properties of the drug are associated with its ability to regulate the redox status, as well as the activity of the nuclear transcription factor NF-*κ*B [106–108].

A progressive decrease in inspiratory capacity (IC) during exercise reflects dynamic hyperinflation and is a significant marker of physical training in patients with COPD. The NAC demonstrated the ability to modify small-diameter airways and the associated processes of pulmonary hyperinflation. In a randomized, double-blind, and placebo-controlled trial by Stav and Raz in 24 patients with moderate and severe stable COPD, there was an increase in IV and forced vital capacity (FVC) after a 6-week course of NAC therapy at a daily dose of 1200 mg. An increase in the ratio of residual lung volume (RV) to the total lung capacity (TLC) was also noted. The distance covered in the exercise test was also significantly greater in the NAC group compared with the placebo group [106–109].

The effect of NAC on the severity of COPD symptoms was evaluated in a systematic review of randomized clinical trials. A statistically significant decrease of the disease symptom intensity in the NAC groups was found in all reviewed studies, in comparison with patients taking the placebo. As a result of a meta-analysis, it was concluded that in 26 out of 100 patients with COPD, NAC therapy reduces the severity of clinical manifestations (number needed to treat(NNT) = 3.8) [110].

Decramer et al. demonstrated that N-acetylcysteine at a dose of 600 mg had no effect on the rate of decline in lung function and the frequency of exacerbations in COPD. However, the analysis in subgroups revealed that the frequency of exacerbations when using NAC was reduced in patients who did not receive inhaled corticosteroids [111].

## 5. Conclusion

NS and OS are multilevel processes, existing and developing in inseparable connection with a number of physiological and pathophysiological processes. They accompany almost all diseases of the respiratory tract, and the study of their subtle mechanisms contributes to improving the quality of diagnosis and the development of therapeutic approaches.

## Figures and Tables

**Table 1:** Respiratory biomarkers and their role in asthma and COPD.

Markers	Specimens	Role in asthma	Role in COPD
FeNO	Exhaled air	Increased in asthma patients and could be used as a potentially valuable tool for assessing the severity of asthma [69].	Elevation in COPD and the association between exacerbated COPD [70].
H_2_O_2_	Exhaled air, EBC	Higher values in uncontrolled asthma [71].	Correlate with COPD health status as measured by the COPD assessment test [72].
8-Isoprostane	EBC, induced sputum	Increased in adult asthmatic and its concentration is related to asthma severity [73].	Increased during exacerbation of COPD [74].
3-NT	EBC, induced sputum	Increased in allergic asthmatics [75].	High levels in COPD [76].
MPO	Induced sputum	Increased in severe asthma patients, associated with neutrophilic inflammation [77, 78].	Increased in stable COPD patients, especially pronounced during exacerbations [79].
EPO	Induced sputum	Elevated amounts of EPO correspond with the increased numbers of eosinophils [80, 81].	
MMPs	Induced sputum	Increased in asthma, associated with airway remodeling [82].	Contribute to the development of emphysema and small airway fibrosis in COPD [83].
MDA	EBC, induced sputum	Increased in acute asthma attacks [84].	Elevated in COPD [76].

Abbreviations: FeNO: fractional exhaled nitric oxide; 3-NT: 3-nitrotyrosine; MPO: myeloperoxidase; EPO: eosinophil peroxidase; MMPs: matrix metalloproteases; MDA: malondialdehyde.
